# Lactic Acid Bacteria and Bifidobacteria with Potential to Design Natural Biofunctional Health-Promoting Dairy Foods

**DOI:** 10.3389/fmicb.2017.00846

**Published:** 2017-05-18

**Authors:** Daniel M. Linares, Carolina Gómez, Erica Renes, José M. Fresno, María E. Tornadijo, R. P. Ross, Catherine Stanton

**Affiliations:** ^1^Teagasc Food Research Centre, MooreparkFermoy, Ireland; ^2^APC Microbiome Institute, University College CorkCork, Ireland; ^3^Department of Food Hygiene and Technology, Faculty of Veterinary Science, University of LeónLeón, Spain

**Keywords:** lactic acid bacteria, bifidobacteria, health, bioactive, probiotic, biofunctional food

## Abstract

Consumer interest in healthy lifestyle and health-promoting natural products is a major driving force for the increasing global demand of biofunctional dairy foods. A number of commercial sources sell synthetic formulations of bioactive substances for use as dietary supplements. However, the bioactive-enrichment of health-oriented foods by naturally occurring microorganisms during dairy fermentation is in increased demand. While participating in milk fermentation, lactic acid bacteria can be exploited *in situ* as microbial sources for naturally enriching dairy products with a broad range of bioactive components that may cover different health aspects. Several of these bioactive metabolites are industrially and economically important, as they are claimed to exert diverse health-promoting activities on the consumer, such as anti-hypertensive, anti-inflammatory, and anti-diabetic, anti-oxidative, immune-modulatory, anti-cholesterolemic, or microbiome modulation. This review aims at discussing the potential of these health-supporting bacteria as starter or adjunct cultures for the elaboration of dairy foods with a broad spectrum of new functional properties and added value.

## Biofunctional Foods

Today foods are not intended to only satisfy hunger and to provide necessary nutrients for humans, but also to prevent nutrition-related diseases and improve consumers’ health (Siro et al., 2008; Gortzi et al., 2015). Increasing consumer demand and interest in obtaining additional benefits from food has stimulated functional foods to emerge on the market, with USA, Europe, and Japan being the dominant markets.

Although there is no unitary accepted definition, functional foods can be described as an ordinary food that has components or ingredients added to provide a specific health benefit, other than a purely nutritional effect. Ideally, functional food refers to an existing traditional food product that is intended to be consumed as part of a normal diet and has a demonstrated added physiological benefit (Siro et al., 2008). Therefore, it could not be in the form of pill or capsule. The concept of biofunctional foods is generally used when this desirable biological, medical, or physiological effect is exerted by microorganisms (Gobbetti et al., 2010). The health benefits of these microorganisms can be exerted either directly through the interactions of ingested live microorganisms with the host (probiotic effect), or indirectly by ingestion of the microbial metabolites synthesized during fermentation (bioactive effect) (Stanton et al., 2005; Gobbetti et al., 2010; Joshi, 2015).

### Probiotic Foods

**FIGURE 1:**
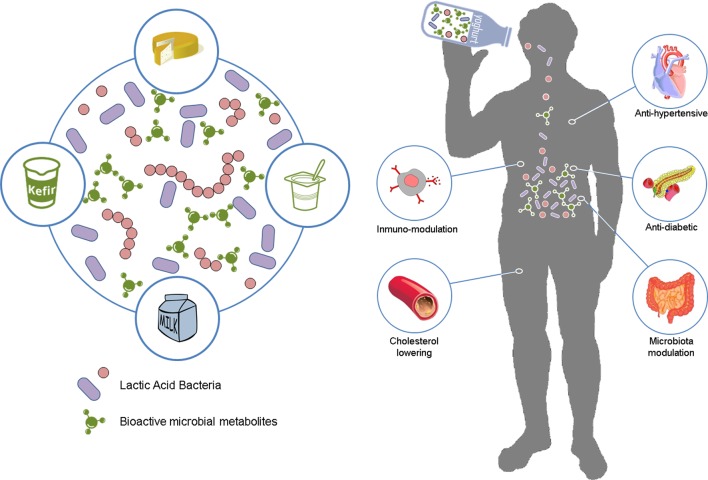
**Beneficial effects resulting from the consumption of biofunctional fermented dairy foods.** Lactic acid bacteria participating in milk fermentation *in situ* release and naturally enrich the fermented dairy product with a broad range of bioactive metabolites. Subsequent ingestion of this product can exert important health-promoting activities on the consumer, such as anti-hypertensive, and anti-diabetic, immune-modulatory, anti-cholesterolemic or microbiome modulation.

Lactic acid bacteria (LAB) have been used to ferment foods for at least 4000 years (Rotar et al., 2007). Although the probiotic concept has expanded more recently, we have been unconsciously ingesting beneficial microbes with traditional fermented foods since ancient times. Fermented foods are the main carriers to deliver probiotics (**Figure 1**). Among them, dairy products (in particular fermented milks and yogurt) are by far the most efficient and widely used (Giraffa, 2012). Cheese is a dairy product which has a good potential for the incorporation of probiotic cultures due to its specific chemical and physical characteristics compared to fermented milks (higher pH value and lower titrable acidity, higher buffering capacity, greater fat content, higher nutrient availability, lower oxygen content, and denser texture). These conditions facilitate survivability of probiotic strains and tolerance to the low pH conditions encountered during gastric transit (Karimi et al., 2011). Utilization of probiotics has been optimized in several cheese varieties such as Cheddar, Gouda, Camembert, Cottage type, white-brined, and traditional cheeses, among others (Araujo et al., 2012; Giraffa, 2012; Martinovic et al., 2016; Oh et al., 2016). Kefir is another milk-fermented product that has health promoting bacteria (Prado et al., 2015). Other non-fermented dairy foods such as low-fat ice cream, chocolate mousse, coconut flan, or infant milk formula have also been supplemented with probiotic strains (Davidson et al., 2000; Aragon-Alegro et al., 2007; Correa et al., 2008; Baglatzi et al., 2016).

Probiotic microorganisms are generally LAB belonging to the species *Lactobacillus acidophilus, L. gasseri, L. helveticus, L. johnsonii, L. (para)casei, L. reuteri, L. plantarum, L. rhamnosus*, and *L. fermentum*, while members of the genus *Bifidobacterium* are also used, e.g., *Bifidobacterium bifidum, B. longum, B. animalis*, and *B. breve* (Tamime et al., 2005; Castro et al., 2015; Linares et al., 2016b). On the basis of the currently available literature, probiotics can balance intestinal microbiota, and thereby regulate proper intestinal function and be effective in the prevention or treatment of several gastrointestinal disorders such as infectious diarrhea, antibiotic-related diarrhea, irritable bowel syndrome or Crohn’s disease (Vanderhoof and Young, 1998) Other examples of health benefits promoted by probiotics supplied via dairy products are inmmunomodulatory effects (*L. casei* CRL431), reduction of serum cholesterol level (*L. reuteri* NCIMB 30242) and antihypertensive effects (*L. plantarum* TENSIA^TM^) (EFSA, 2011; Jones et al., 2012; Aragon et al., 2014).

Probiotics are defined as ‘live micro-organisms, which when consumed in adequate amounts confer a health benefit on the host’ (FAO/WHO, 2001). However, regarding probiotic foods, some considerations are of paramount importance. Firstly, effective levels of the live probiotic in the corresponding food matrix at the time of ingestion are required. In this regard, the minimum effective dose which affects the intestinal environment and provides beneficial effects on human health is considered to be 10^6^–10^9^ live microbial cells per day, although this depends on the particular strain and foodstuff (Williams, 2010; Karimi et al., 2012; Watson and Preedy, 2015). Since probiotics show beneficial health effects on the host once consumed, another precondition for a bacterial strain to be called probiotic is the ability to survive and colonize (at least transiently) the gastrointestinal tract (GIT), which is in part helped by the buffering capacity of the food matrix. In some particular cases, bacterial viability may not be strictly required. As an example, inactivated and dead *L. rhamnosus* GG cells can maintain immunological and health-promoting effects (Ghadimi et al., 2008; Lopez et al., 2008).

### Bioactive Compounds Derived from Microbes

Microorganisms involved in dairy fermentations can produce biologically active molecules and enzymes, giving the final food product an additional health value. Unlike the probiotic concept (the bacteria must be ingested alive and produce the beneficial metabolite in the body), the biofunctional concept is generally used when the healthy metabolite emerge in the food product itself during the fermentation process as a consequence of the bacterial metabolic activity. Consequently, the bacteria can act as a microbial factory to enrich foodstuff, for which bacterial viability through the GIT or during the product storage is not absolutely required (Farnworth and Champagne, 2015). The main bioactive compounds produced by LAB during dairy fermentation are vitamins, gamma-aminobutyric acid, bioactive peptides, bacteriocins, enzymes, conjugated linoleic acid, and exopolysaccharides (**Table 1**).

**Table 1:** Some strains of lactic acid bacteria, bifidobacteria and propionibacteria with potential to biosynthesize health-promoting compounds in fermented dairy products.

Bioactive	Producer strain	Food Product	Health effect	Reference
**Thiamine (B_1_)/Riboflavin (B_2_)**	*Lactobacillus casei* KNE-1	Fermented milk	Vitamin enrichment	Drywien et al., 2015
	*Bifidobacterium infantis* CCRC14633	Fermented soymilk	Vitamin enrichment	Tamime, 2006
	*Bifidobacterium longum* B6	Fermented soymilk	Vitamin enrichment	Tamime, 2006
	*Lactobacillus plantarum* CRL 2130	Fermented soymilk	Vitamin enrichment	Levit et al., 2016
**Biotin (Vitamin B_7_)**	*Lactobacillus helveticus MTCC5463*	Fermented milk	Vitamin enrichment	Patel et al., 2013
**Cobalamin (Vitamin B_12_)**	*Propionibacterium freudenreichii*	Kefir	Vitamin enrichment	Van Wyk et al., 2011
	*Bifidobacterium animalis* Bb12	Fermented milk	Vitamin enrichment	Patel et al., 2013
	*Lactobacillus reuteri* ZJ03	Soy-yogurt	Vitamin enrichment	Gu et al., 2015
**Folic acid (Vitamin B_9_)**	*Streptococcus thermophilus* CRL803/CRL415	Yogurt	Vitamin enrichment	Laiño et al., 2013
	*Lactobacillus bulgaricus* CRL871	Yogurt	Vitamin enrichment	Laiño et al., 2013
	*Bifidobacterium lactis* CSCC5127	Fermented milk	Vitamin enrichment	Crittenden et al., 2003
	*Bifidobacterium infantis* CSCC5187	Fermented milk	Vitamin enrichment	Crittenden et al., 2003
	*Bifidobacterium breve* CSCC5181	Fermented milk	Vitamin enrichment	Crittenden et al., 2003
	*Lactobacillus amylovorus* CRL887	Fermented milk	Vitamin enrichment	Laiño et al., 2014
**GABA**	*Lactobacillus casei* Shirota	Fermented milk	Antidiabetic, blood pressure	Inoue et al., 2003
	*Streptococcus salivarius* fmb5	Fermented milk	Antidiabetic, blood pressure	Chen et al., 2016
	*Lactobacillus plantarum* NDC75017	Fermented milk	Antidiabetic, blood pressure	Shan et al., 2015
	*Lactobacillus brevis* OPY-1	Fermented soya milk	Antidiabetic, blood pressure	Park and Oh, 2007
	*Streptococcus thermophilus* APC151	Yogurt	Antidiabetic, blood pressure	Linares et al., 2016a
**Bioactive peptides**	*Lactobacillus helveticus* Evolus^®^	Fermented milk	Anti-hypertensive	EFSA, 2008
	*Lactobacillus helveticus/S. cerevisiae* (Calpis^TM^)	Fermented milk	Anti-hypertensive	Dziuba and Dziuba, 2014
	*Lactobacillus bulgaricus* LB340	Fermented milk/yogurt	Anti-hypertensive, Immunomodulatory	Qian et al., 2011
**Bacteriocins**	*Lactococcus lactis* CNRZ150/TAB50	Camembert/Semihard cheese	Pathogen inhibition	Arques et al., 2015
	*Lactococcus lactis* DPC3147	Cheddar cheese	Pathogen inhibition	Ross et al., 1999
	*Lactobacillus acidophilus* CH5	Yogurt	Pathogen inhibition	Ahmed et al., 2010
	*Pediococcus acidilactici* CHOOZIT^TM^	Cheddar/Semihard cheese	Pathogen inhibition	Arques et al., 2015
	*Lactobacillus plantarum* WHE92	Munster cheese	Pathogen inhibition	Arques et al., 2015
**Conjugated linoleic acid**	*Lactococcus lactis* CI4b	Cheddar cheese	Cholesterol lowering	Mohan et al., 2013
	*Lactobacillus rhamnosus* C14, *Lactobacillus casei* CRL431, *Streptococcus thermophilus* CRL728, *Bifidobacterium bifidum* CRL1399	Buffalo cheese	Cholesterol lowering	Van Nieuwenhove et al., 2007a
	*Lactococcus lactis* LMG, *Lactobacillus acidophilus* Lac1, *Lactobacillus plantarum -*2, *Bifidobacterium animalis* Bb12	Fermented buffalo milk	Cholesterol lowering	Van Nieuwenhove et al., 2007b
	*Lactobacillus bulgaricus* LB430*/Streptococcus thermophilus* TA040	Yogurt	Cholesterol lowering	Sosa-Castañeda et al., 2015
**Exopolysaccharides**	*Lactobacillus bulgaricus* OLL1073R-1	Yogurt	Immunostimulatory	Makino et al., 2016
	*Lactobacillus mucosae* DPC 6426	Yogurt/Cheddar cheese	Hypocholesterolemic	Ryan et al., 2015
	*Propionibacterium freudenreichii* KG15/KG6	Turkish cheese	Microbiota modulation	Darilmaz and Gumustekin, 2012
	*Lactococcus lactis* SMQ-461	Cheddar cheese	Microbiota modulation	Dabour et al., 2005
	*Lactobacillus plantarum* YW11	Kefir	Microbiota modulation	Wang et al., 2015
	*Bifidobacterium longum* CCUG52486	Yogurt	Immune modulation	Prasannaa et al., 2013
	*Streptococcus thermophilus* zlwTM11	Yogurt	Microbiota modulation	Han et al., 2016,
	*Streptococcus thermophilus* FD-DVSST-BODY3	Fermented ice-cream	Microbiota modulation	Dertli et al., 2016


#### Vitamins

There are 13 vitamins that must be obtained exogenously due to the inability of humans to synthesize them; thereby they are essential nutrients in the human diet, and although in small amounts, a daily requirement is necessary to prevent deficiencies. Although most vitamins are present in a variety of foods, human vitamin deficiencies still occur in many countries, mainly because of malnutrition, not only as a result of insufficient food intake but also because of unbalanced diets (LeBlanc et al., 2011).

Although milk contains many vitamins fermentation by LAB and bifidobacteria constitute an effective way to increase vitamin levels in milk (Laiño et al., 2013). Some bacterial strains included in the genera *Lactobacillus* and *Bifidobacterium* can provide an additional source of B vitamins (thiamine, riboflavin, cobalamin, folate, and biotin) during dairy fermentation. Deficiencies in riboflavin (vitamin B_2_) or thiamine (vitamin B_1_) can lead to both liver and skin disorders and alterations in brain glucose metabolism, respectively (Russo et al., 2014). In this regard, *L. casei* KNE-1 has been shown to produce thiamine and riboflavin in fermented milk drinks (Drywien et al., 2015). *B. infantis* CCRC14633 and *B. longum* B6 strains have been reported to produce riboflavin and thiamine during soymilk fermentation (Tamime, 2006). It was recently indicated that soymilk fermented by the riboflavin-producing strain *L. plantarum* CRL2130 was able to prevent ariboflavinosis and experimental colitis in a murine model (Juarez del Valle et al., 2016; Levit et al., 2016). Some propionibacteria can produce cobalamin, folic acid, and biotin (Hugenholtz et al., 2002).

Folate (vitamin B_9_) is involved in several vital processes and its deficiency is generally linked to neural tube defects, certain forms of cancer, poor cognitive performance and coronary heart diseases. Even though vitamins are widely present in foods, the prevalence of folate deficiency -especially among women of child bearing age- is a growing concern and thereby folate fortification programs have been implemented in various countries (Divya and Nampoothiri, 2015). Rather than incorporating synthetic folate, foods can be naturally fortified with folate synthesized by LAB and bifidobacteria during manufacture of fermented foods (Lin and Young, 2000; Saubade et al., 2016). The strains *Streptococcus thermophilus* CRL803/CRL415 and *L. bulgaricus* CRL871 were reported to be suitable for the elaboration of yogurt naturally bio-enriched in this vitamin (Laiño et al., 2013). High folate concentration (up to 150 μg/l) can be reached in yogurt as a result of the ability of *S. thermophilus* to produce this vitamin (Hugenholtz et al., 2002). Among bifidobacteria, *B. catenulatum* ATCC 27539 was shown to produce high levels of folate *in vitro* (D’Aimmo et al., 2012), and *B. lactis* CSCC5127, *B. infantis* CSCC5187, and *B. breve* CSCC5181 strains increased folate concentration during fermentation of reconstituted skim milk (Crittenden et al., 2003). Similarly, *L. amylovorus* CRL887 can be used for natural folate bio-enrichment of fermented milk (Laiño et al., 2014).

The deficiency of cobalamin (vitamin B_12_) can be common, particularly in vegetarians who avoid ingestion of animal protein and use soymilk as an alternative to dairy milk (Gu et al., 2015). Animals, plants and fungi are incapable of producing this vitamin, and hence, it is exclusively produced by microorganisms (LeBlanc et al., 2011). It has been demonstrated that cobalamin can be synthesized by some bacteria such as *L. reuteri* ZJ03, *Propionibacterium freudenreichii*, *B. animalis* Bb12 in soy-yogurt, kefir and fermented milk, respectively (Van Wyk et al., 2011; Patel et al., 2013, Gu et al., 2015; Moslemi et al., 2016). Microorganisms can biosynthesize two different isoforms, the vitamin and the pseudovitamin. For example, in a recent work, the production of vitamin and pseudovitamin B_12_ by *P. freudenreichii* was quantified specifically and shows that at the initial phase of the fermentation both isoforms are biosynthesized at similar levels; however, by the end of the fermentation the pseudovitamin is not detected, most likely because it is converted to the vitamin form (Deptula et al., 2017). It seems crucial to differentiate between the two isoforms of this vitamin, as the transporter protein in the human GIT has very low affinity for the pseudovitamin, making it un-available to humans (Varmanen et al., 2016).

Biotin (vitamin B_7_) deficiency can be caused by inadequate dietary intake or some inborn genetic disorders that affect its metabolism. Subclinical deficiency can cause mild symptoms, such as hair thinning or skin rash typically on the face. Biotin can be synthesized by some LAB in dairy products, e.g., *L. helveticus* MTCC 5463 increased biotin content in fermented milks (Patel et al., 2013). Some propionibacteria can also produce biotin (Hugenholtz et al., 2002).

Vitamin K is an important promoter of bone and cardiovascular health. It has been associated with the inhibition of arterial calcification and stiffening, and the reduction of vascular risk. This vitamin is nearly non-existent in junk food, with little being consumed even in a healthy Western diet (Maresz, 2015). Its deficiency has been implicated in several clinical ailments such as intracranial hemorrhage in newborn infants and possible bone fracture resulting from osteoporosis (LeBlanc et al., 2011). Vitamin K occurs in two forms: *firstly*, phylloquinone (vitamin K_1_), which is present in green plants, and *secondly*, menaquinone (vitamin K_2_), which is produced by some intestinal bacteria (LeBlanc et al., 2011). Menaquinone can be biosynthesized by some LAB species (mainly *Lactococcus lactis*) commonly used in industrial fermentation of cheese, buttermilk, sour cream, cottage cheese, and kefir (Walther et al., 2013). Other LAB have been screened for the ability to produce menaquinone; these included strains from the genera *Lactococcus*, *Bifidobacterium*, *Leuconostoc*, and *Streptococcus* (Morishita et al., 1999). Although the MK forms are ubiquitous in bacteria, it should be noted that some genera such as *Lactobacillus* have lost the functional ability to produce them (Lechardeur et al., 2011; Walther et al., 2013).

#### Gamma-Aminobutyric Acid

Gamma-aminobutyric acid (GABA) is the main inhibitory neurotransmitter of the central nervous system (CNS). Several important physiological functions of GABA have been characterized, such as neurotransmission, induction of hypotension, diuretic effects, antidiabetic, relaxing and tranquilizer effects (Inoue et al., 2003; Marques et al., 2016). In fact, some GABA_A_-receptor agonist drugs (e.g., benzodiazepines) are important pharmacological agents used for clinical treatment of anxiety (Foster and Kemp, 2006).

Gamma-aminobutyric acid is biosynthesized through α-decarboxylation of glutamate, an enzymatic conversion which is catalyzed by glutamate decarboxylase (GAD) (Tajabadi et al., 2015). Several food-grade LAB have been reported to exhibit GABA-producing ability. Among them, most of the GABA-producing strains are lactobacilli (*L. brevis*, *L. paracasei*, *L. delbrueckii, L. buchneri*, *L. plantarum*, *L. helveticus*), *Streptococcus thermophilus*, and *Lactococcus lactis* (Li and Cao, 2010; Dhakal et al., 2012). Some, *Bifidobacterium* spp. were also reported to produce GABA, although with lower capacity than LAB (Park et al., 2005; Barrett et al., 2012).

Some fermented dairy products enriched in GABA using GABA-producing LAB as starters have been developed. The strains *L. casei* Shirota, *S. salivarius* fmb5 and *L. plantarum* NDC75017 were utilized to ferment and enrich milk in GABA (Inoue et al., 2003; Shan et al., 2015; Chen et al., 2016). More recently, yogurt enriched with 2 mg GABA/ml was produced using the strain *S. thermophilus* APC151 (Linares et al., 2016a, 2017). Also, fermented soya milk (using *L. brevis* OPY-1 as source of GABA) (Park and Oh, 2007), or cheese (*Lactococcus lactis* as source of GABA) (Nomura et al., 1998; Pouliot-Mathieu et al., 2013) have been produced. Thus, GABA has potential as a health-promoting bioactive component in foods (Li and Cao, 2010).

#### Bioactive Peptides

During milk fermentation, LAB, making use of their proteolytic system can transform milk proteins into biologically active peptides. These peptides can exert a wide range of effects, such as antimicrobial, antihypertensive, antithrombotic, immunomodulatory, and antioxidative (LeBlanc et al., 2002; Nongonierma and FitzGerald, 2015). The most studied mechanism of bioactive peptides is the antihypertensive action displayed by the inhibition of the angiotensin-I-converting enzyme (ACE; peptidyldipeptide hydrolase, EC 3.4.15.1) which regulates blood pressure (Fernandez et al., 2015). ACE inhibitory peptides have been isolated from a variety of fermented dairy products including cheese, fermented milks and yogurt (Fitzgerald and Murray, 2006; Pritchard et al., 2010). The best known ACE-inhibitory biopeptides, Val-Pro-Pro (VPP) and Ile-Pro-Pro (IPP), have been identified in milk fermented by *L. helveticus* (Slattery et al., 2010). In addition, other dairy starter cultures industrially used in the manufacture of fermented dairy products (e.g., *L. helveticus, L. delbrueckii* ssp. *bulgaricus, L. plantarum, L. rhamnosus, L. acidophilus, Lactococcus Lactis*, or *S. thermophilus*) can generate bioactive peptides (Hajirostamloo, 2010; Hafeez et al., 2014). Other ACE-inhibitory peptides such as β-casein f(72-81), Ser-Lys-Val-Tyr-Pro-Phe-Pro-Gly-Pro-Ile (SLVYPFPGPI) have been produced by *L. delbrueckii* ssp. *bulgaricus* LB340 in fermented milk (Qian et al., 2011).

On an industrial scale, two fermented milk products with antihypertensive claims, Calpis^TM^ and Evolus^®^, have been tested extensively in rats and in clinical trials, and are commercialized as functional foods (Dziuba and Dziuba, 2014). Evolus^®^ is available in the market as a *L. helveticus* fermented milk -produced in Finland- proven to decrease the systolic blood pressure in hypertensive subjects due to the actions of *L. helveticus* bioactive peptides (EFSA, 2008). Calpis^TM^ is defined as a milk product marketed in Japan (Calpis Co. Ltd.) with antihypertensive properties. It is prepared by fermenting skimmed milk with *L. helveticus* and *Saccharomyces cerevisiae*, which produce VPP and IPP peptides from β-casein and κ-casein (Dziuba and Dziuba, 2014).

#### Bacteriocins

Bacteriocins are ribosomally synthesized antimicrobial peptides produced by a particular bacterium that are active against other competitor bacteria; thereby they constitute an important part of the microbial defense system (Nes et al., 2007). Such bacteriocin-producing strains may offer potential as an alternative to antibiotics, and may be useful as a means of controlling pathogen carriage, therefore being highly suitable as microbial food additives (Cotter et al., 2013) (**Table 2**). Bacteriocins from LAB have attracted much interest because they are frequently produced by commercially useful strains that are generally regarded as safe (GRAS) for human consumption (Nes et al., 2007). These antimicrobial molecules are among the beneficial peptides intrinsically synthesized by some LAB during milk fermentation and they have been traditionally used as naturally produced food biopreservatives. In addition, they may function in the GIT as potential natural biotherapeutic agents facilitating the competition of probiotic strains and/or inhibition of pathogens; thereby they are potential contributors to the microbiota balance and human health (Dobson et al., 2012).

**Table 2:** Characteristic aspects of bacteriocins compared to conventional antibiotics (Adapted from Cleveland et al., 2001).

	Bacteriocins	Antibiotics
Application	Foods	Clinical
Bioactivity spectra	Mostly narrow	Mostly broad
Bioactivity intensity	nM – μM	μM – mM
Biosynthesis	Ribosomal	Secondary metabolite
Proteolytic degradability	High	None
Thermostability	High	Low
Activity pH range	Wide	Narrow
Target cell resistance development	Adaptation through changes in cell membrane composition	Genetically transferable determinant that inactivates the active compound
Mode of action	Generally, pore formation.	Cell membrane or intercellular targets, inhibition of cell wall biosynthesis
Toxicity in eukaryotic cells	None known	Present


Nisin is the most well-known bacteriocin used as food preservative due to its antibacterial effect against *Listeria*, clostridia spores and LAB associated to spoilage. Nisin has been approved as a food additive (E234) in the European Union according to Directive 95/2/EC (EC, 1995) in the following products: semolina and tapioca puddings (3 mg/kg); ripened and processed cheese (12.5 mg/kg), clotted cream (10 mg/kg), and Mascarpone cheese (10 mg/kg). It is also permitted in over 40 countries world-wide including USA, Australia, South Africa, Russia, and India for use as an antimicrobial agent in a variety of food products (EFSA, 2006). Nisin-containing Camembert and semihard cheeses with prolonged shelf-life were made using *Lactococcus lactis* (strains CNRZ150 or TAB50, respectively) as nisin producers (Arques et al., 2015). Apart from nisin, plantaricins are very well-known bacteriocins. For example, plantaricin C is a broad spectrum bacteriocin produced by *L. plantarum*, isolated from ripening cheese (Gonzalez et al., 1994). Plantaricins have been reported to produce an immunomodulatory effect on dendritic cells (Meijerink et al., 2010). However, bacteriocins other than nisin have so far only few and limited authorized uses in foods (Yang et al., 2015). Consequently, the use of bacteriocin-producing bacteria as starter culture for *in situ* biosynthesis during milk fermentation becomes an effective alternative strategy to incorporate bacteriocins in dairy foods. Similarly, the lacticin 3147-producing strain *Lactococcus lactis* DPC3147 used as a protective culture in Cheddar cheese reduced numbers of *Listeria monocytogenes* to <10 cfu/g within 5 days at 4°C (Ross et al., 1999; Chen and Hoover, 2006). Other bacterial species such as *L. acidophilus* can be added as an adjunct in many food fermentation processes to contribute to bacteriocin production and food preservation (Anjum et al., 2014). Other LAB strains such as *L. plantarum* WHE92 used as adjunct to the starter culture reduced *Listeria monocytogenes*, *Listeria innocua*, and *Escherichia coli* O157:H7 counts in cheese as a consequence of the production of plantaricin (Arques et al., 2015). Using a similar concept, Danisco developed a freeze-dried culture of *Pediococcus acidilactici* (marketed as CHOOZIT Flav 43) for use as a bacteriocin-producer adjunct in Cheddar and semihard cheeses (Mills et al., 2011).

Studies of the direct impact of dairy foods containing bacteriocins on human health and microbiome are still limited. *In vivo* antimicrobial activity of nisin and lacticin 3147 has been recently demonstrated in a murine infection model. A nisin-producing *Lactococcus lactis* CHCC5826 modified the microbiota composition of human microbiota-associated rats increasing bifidobacteria levels and decreasing *Enterococcus/Streptococcus* populations. Lacticin 3147 has the potential to be employed in the treatment of *Clostridium difficile* diarrhea and to eliminate the pathogen when added to an anaerobic fecal fermentation (Arques et al., 2015).

#### Enzymes

Lactic acid bacteria associated to dairy fermentations possess enzymes which can be produced *in situ* during fermentation of dairy foods and have bioactive potential on the consumer. Examples are hydrolytic enzymes that may exert potential synergistic effects on digestion and alleviate symptoms of intestinal malabsorption (Patel et al., 2013). A well-known example is the β-galactosidase activity, which can achieve lactose degradation and thereby improve health and reduce symptoms of lactose intolerant consumers. Yogurt and other conventional starter cultures and probiotic bacteria in fermented and unfermented milk products improve lactose digestion and alleviate symptoms of intolerance in lactose malabsorbers. These beneficial effects are due to microbial β-galactosidase (de Vrese et al., 2001).

#### Conjugated Linoleic Acid

Conjugated linoleic acid (CLA) is a polyunsaturated fatty acid (PUFA) that can be biosynthesized by LAB and bifidobacteria through bioconversion of linoleic acid (LA; *cis*-9,*cis*-12 C18:2). The two isomers that have been shown to have bioactive potential are *cis*-9,*trans*-11 (*c*9,*t*11) and *trans*-10,*cis*-12 (*t*10,*c*12). The health-promoting properties of CLA include anticarcinogenic, antiatherogenic, anti-inflammatory, and antidiabetic activity, as well as the ability to reduce body fat (Sosa-Castañeda et al., 2015). Although it is a native component of milk, the amount consumed in foods is far from that required in order to obtain desired beneficial effects. Thus, increasing the CLA content in dairy foods through milk fermentation with specific LAB offers a promising alternative. An effective way to increase CLA uptake in humans is to increase CLA levels in dairy products by using strains with high production potential (Lee et al., 2007). A number of food-grade LAB and bifidobacteria were reported to produce CLA in milk products (Sosa-Castañeda et al., 2015; Yang et al., 2015), as is the case of *Lactococcus lactis* LMG, *L. rhamnosus* C14, *L. casei* CRL431, *L. acidophilus* Lac1, *L. plantarum*-2, *B. bifidum* CRL1399 and *B. animalis* Bb12 (Van Nieuwenhove et al., 2007a; Florence et al., 2009). Some of these strains were also used as adjunct cultures for the manufacture of high CLA-content buffalo cheese (Van Nieuwenhove et al., 2007b). The CLA-producing starter culture of *Lactococcus lactis* CI4b enhanced levels of total CLA in Cheddar cheese (Mohan et al., 2013). Similarly, *L. bulgaricus* LB430 and *S. thermophilus* TA040 are suitable for production of CLA-enriched yogurt (Florence et al., 2009).

In addition, it has been shown that specific microorganisms such as *L. plantarum* PL60 or *B. breve* NCIMB702258, are able to produce CLA following dietary administration in animal models (Wall et al., 2009, 2012) and following the administration as a freeze-dried product in humans (Lee and Lee, 2009). Thus, intestinal CLA production by bacteria may contribute to enhance CLA supply in addition to the CLA provided by the producing strain in fermented milks during the manufacture (Teran et al., 2015).

#### Exopolysaccharides

Exopolysaccharides (EPS) are complex extracellular carbohydrate polymers that can be produced by some LAB *in situ* during dairy fermentations. Some of them promote selective growth of bifidobacteria, thus playing a role on the host microbiota and immune system (Fernandez et al., 2015; Salazar et al., 2016). In this regard, EPS derived from yogurt fermented with *L. delbrueckii* ssp. *bulgaricus* OLL1073R-1 exerted immune-stimulatory effects in mice (Makino et al., 2016). Yogurt, Swiss-type, and Cheddar cheeses represent suitable food matrices for the delivery of the hypocholesterolemic EPS-producer strain *L. mucosae* DPC 6426 (Ryan et al., 2015). Other microorganisms with potential to produce EPS in cheese are *P. freudenreichii* KG15/KG6, *Lactococcus lactis* SMQ-461 or *S. thermophilus* MR-1C (Dabour et al., 2005; Darilmaz and Gumustekin, 2012). Significant levels of EPS can also be produced in kefir by *L. plantarum* YW11 (Wang et al., 2015). Recently, EPS produced by bifidobacteria have attracted the attention due to their immune modulation capability (Hidalgo-Cantabrana et al., 2012).

Exopolysaccharides can also improve the quality, sensory and rheological properties of the final food product, which in many cases results in a reduction of the amount of chemical stabilizers required, thus favoring a more natural product. For example, strains of *B. longum* subsp. *infantis* CCUG 52486 and *S. thermophilus* were suitable to produce yogurt and fermented ice-cream with improved viscosity and texture and reduced syneresis as a consequence of their high EPS production (Prasannaa et al., 2013; Han et al., 2016; Dertli et al., 2016).

## Regulatory Aspects

At present, the status of probiotic-based products is full of ambiguities because various regulatory agencies in different countries are defining and categorizing probiotics differently. Despite the emerging interest of consumers toward probiotics and functional foods, in Europe the regulatory framework is still not harmonized and no health claim for probiotics alone (except yogurt starters) has been approved. Paradoxically, probiotics or bioactive bacteria have been introduced into the market as dietary supplements or natural health products (capsules, tablets, and powders) (Arora and Baldi, 2015). Japan was the very first global jurisdiction for implementing a regulatory system for functional foods and nutraceuticals in 1991, and is currently acting as global market leader where probiotics are available as both foods and drugs. The government has designated Foods for Specific Health Uses (FOSHU), which classifies health claims into different subcategories (gastrointestinal health, cholesterol moderation, hypertension moderation, lipid metabolism moderation, sugar absorption moderation, mineral absorption, and bone and tooth health). In China, State Food and Drug Administration (SFDA) has regulated all health foods including functional foods and nutraceuticals, and a well-developed market for functional foods is established (Arora and Baldi, 2015). Currently USA is regulating probiotics as a variety of products as per their intended usage and regulatory bodies are Dietary Supplement Health and Education Act (DSHEA) and Food and Drug administration (FDA). Dietary supplements are considered as ‘foods’ and are regulated by DSHEA and do not need FDA approval before being marketed. However, probiotics and dietary supplements containing a new dietary ingredient without a marketing history are regulated by FDA. In conclusion, a harmonized categorization of probiotics and functional foods may help to regulate these products whenever solid clinical documentation is available to support any health effects and health messages in human subjects. The appropriate level of evidence for determining a health benefit for probiotics should always be put ahead of commercial and labeling industrial interests.

## Challenges in Industry and Concluding Remarks

A goal of the dairy industry is to develop novel dairy products with increased nutritional and/or health promoting properties. Food-grade bacteria have the potential to fortify fermented dairy food products with bioactive metabolites by a natural process, thereby reducing the need for fortification with costly chemically synthesized supplements. Nowadays, a number of commercial sources have available synthetic formulations of bioactive substances for their use as a dietary supplement. The use of health-supporting bacteria for naturally enriching dairy foods with bioactives could be a suitable alternative to food fortification with chemical formulations.

The starter cultures must be carefully selected, since the ability of microbial cultures to produce bioactive metabolites is generally a strain-dependent trait and varies considerably among strains within the same species. The yield of bioactive synthesis and the concentration of such compound in dairy products is another critical strain-dependent factor. In this regard, the dose of bioactives ingested with the corresponding food product should remain over the minimum required to meet the human requirements and/or have the claimed therapeutic level on the consumer, according to existing clinical recommendations and studies. An open question when using co-cultures or strain combinations is their interaction in terms of nutrient availability, bacterial growth, as well as the bioactive production yield. In some cases, metabolites (i.e., vitamins etc.) produced by one of the strains could be consumed by the other strains, thus decreasing the final content in food.

Generally, the biosynthetic pathways are genetically encoded. In this regard, the increasing availability of bacterial genome sequences over the last decade has provided a major contribution to the knowledge about microbial production of bioactive molecules. However, the presence of the genes required for the biosynthesis of a particular biomolecule should not be assumed as synonym of metabolite production. Typical exceptions to the correlation genotype-phenotype occur when the genes are not active or when the metabolite is intracellularly biosynthesized and a release system is lacking. This is indeed one of the major bottlenecks during biosynthesis of some vitamins that needs to be overcome through the use of alternative strategies such as autolytic mutants and metabolic engineering (Basavanna and Prapulla, 2013).

Consideration should also be given by manufacturers to the optimum conditions for bioactive compound biosynthesis by LAB during technological processes. The content and activity of a bioactive compound in the dairy fermented foodstuffs is the result of the type of food matrix, the individual bacterial strain properties as well as the processing conditions and storage time. In this regard, it should be noted that the high bioactive biosynthetic rates observed in culture media might not always be extrapolated to dairy products. Therefore, factors such as optimal temperature for microbial growth and viability, food composition or bioactive stability and shelf-life in the final foodstuff are of paramount importance to reach the maximum concentration and activity in the final product.

Overall, the current review updates knowledge about LAB, bifidobacteria and propionibacteria with potential to enrich dairy food products with health-promoting bio-metabolites. Promising applications at commercial level emerge; however, adequate selection of strains is vital to increase the concentration and bioavailability of such biomolecules in fermented foods. The use of LAB and bifidobacteria able to synthesize bioactive components in fermented foods could help to provide these compounds in foods, this being in compliance with current regulatory rules.

## Author Contributions

DL provided the general concept, and drafted part of the manuscript. CG and ER wrote part of the manuscript. All authors revised and approved the manuscript.

## Conflict of Interest Statement

The authors declare that the research was conducted in the absence of any commercial or financial relationships that could be construed as a potential conflict of interest.
